# Piezo1, the new actor in cell volume regulation

**DOI:** 10.1007/s00424-024-02951-y

**Published:** 2024-04-06

**Authors:** A. Michelucci, L. Catacuzzeno

**Affiliations:** Department of Chemistry, Biology and Biotechnology, University of Perugia, 06123 Perugia, Italy

**Keywords:** Cell death, K_Ca_ channels, Migration, Piezo1, Regulatory volume decrease, VRAC

## Abstract

All animal cells control their volume through a complex set of mechanisms, both to counteract osmotic perturbations of the environment and to enable numerous vital biological processes, such as proliferation, apoptosis, and migration. The ability of cells to adjust their volume depends on the activity of ion channels and transporters which, by moving K^+^, Na^+^, and Cl^−^ ions across the plasma membrane, generate the osmotic gradient that drives water in and out of the cell. In 2010, Patapoutian’s group identified a small family of evolutionarily conserved, Ca^2+^-permeable mechanosensitive channels, Piezo1 and Piezo2, as essential components of the mechanically activated current that mediates mechanotransduction in vertebrates. Piezo1 is expressed in several tissues and its opening is promoted by a wide range of mechanical stimuli, including membrane stretch/deformation and osmotic stress. Piezo1-mediated Ca^2+^ influx is used by the cell to convert mechanical forces into cytosolic Ca^2+^ signals that control diverse cellular functions such as migration and cell death, both dependent on changes in cell volume and shape. The crucial role of Piezo1 in the regulation of cell volume was first demonstrated in erythrocytes, which need to reduce their volume to pass through narrow capillaries. In HEK293 cells, increased expression of Piezo1 was found to enhance the regulatory volume decrease (RVD), the process whereby the cell re-establishes its original volume after osmotic shock-induced swelling, and it does so through Ca^2+^-dependent modulation of the volume-regulated anion channels. More recently we reported that Piezo1 controls the RVD in glioblastoma cells via the modulation of Ca^2+^-activated K^+^ channels. To date, however, the mechanisms through which this mechanosensitive channel controls cell volume and maintains its homeostasis have been poorly investigated and are still far from being understood. The present review aims to provide a broad overview of the literature discussing the recent advances on this topic.

## Introduction

### Cell volume regulation

The ability of cells to finely regulate their volume is essential for maintaining cell function and viability. Cell volume, which depends on the cell’s water content, is ultimately determined by the cytoplasmic osmotic force, relative to the outside. Therefore, when the internal osmolarity increases compared to the external one, water enters the cell and quite rapidly the cell volume increases (swelling). The opposite—shrinkage—occurs when the internal osmolarity falls below that of the extracellular environment. Plasma membrane channels and active transporters play the main role in regulating cell volume, as they mediate the passage of electrolytes in and out of the cell and actively create the osmotic gradient necessary for the net movement of water.

**Fig. 1:**
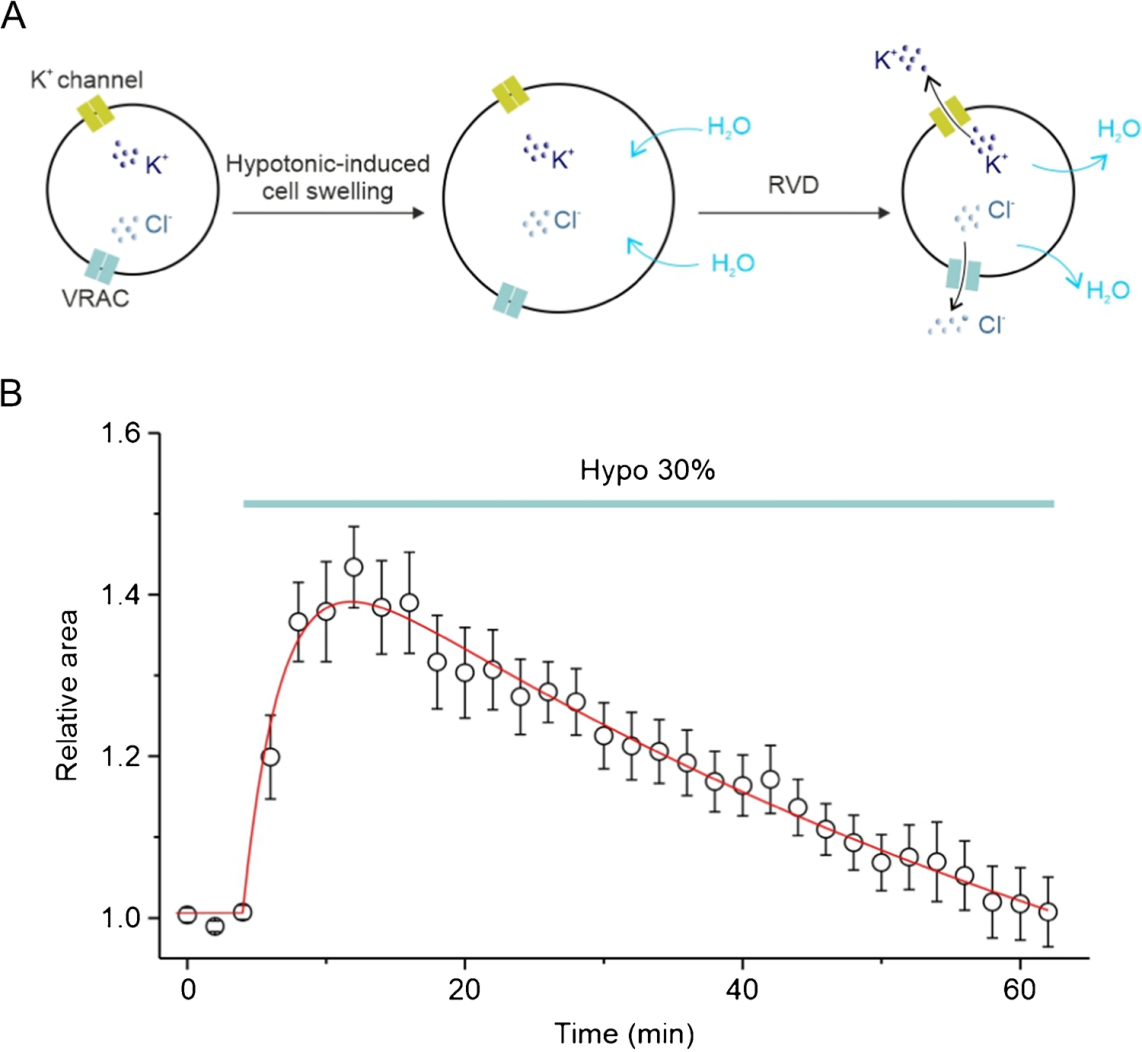
General aspects of cell volume regulation (RVD). **A** Schematic representation of RVD. Upon exposure to extracellular hypotonic stimulus, cells undergo a rapid swelling due to osmotic influx of water. Cell swelling leads to the opening of both Cl^−^ and K^+^ channels, allowing net efflux of KCl that drives the osmotic loss of water, which in turn re-establish the original cell volume. Whereas VRAC is largely recognized as the main channel involved in the transport of Cl^−^ ions in virtually all animal cells, the nature of K^+^ channels is less known and can vary significantly depending on the cell type. **B** Representative time course of RVD evaluated from changes of the relative cell area following application of 30% hypotonic solution (Hypo 30%, cyan bar). Cell area was assessed by video imaging using contrast-phase microscopy. Data are shown as mean ± SEM

Regulatory volume decrease (RVD) is an evolutionarily conserved process, used by animal cells to restore their normal volume in the event of osmotic shock-induced swelling. RVD plays an important role in many physiological processes, including the prevention of necrotic cell death induced by persistent cell swelling. In addition, the ability of cells to locally reduce their volume and change their shape is crucial for processes such as migration. RVD is mainly mediated by the concerted activity of ion channels that mediate the passage of Cl^−^ and K^+^ ions. The net efflux of KCl, upon cell swelling, is used by the cell to create the osmotic gradient to extrude water and recover its volume (Fig. 1).

#### Ion channels involved in cell volume regulation

The volume-regulated anion channel (VRAC), a heteromeric protein formed by five subunits encoded by the *lrrc8* gene family [131, 165], which mediates the swelling-activated Cl^−^ current (I_Cl,swell_) upon hypotonic cell swelling, was identified as the common and principal channel responsible for the Cl^−^ transport during RVD in virtually all vertebrate cells [24, 55, 75, 118, 141, 145, 146]. By contrast, the nature of K^+^ channels that mediate K^+^ efflux is much more elusive and can vary in different cell models. Among them, stretch-activated K^+^ channels [47, 54, 139, 163], voltage-dependent K^+^ channels [20, 45, 52, 92], and Ca^2+^-activated K^+^ (K_Ca_) channels of large, intermediate, and small conductance (BK, IK, and SK, respectively) [126, 166, 169], have been reported to play key roles in volume regulation in many cell types.

In addition to Cl^−^ and K^+^ channels, whose activity is strictly related to the generation of the osmotic gradients necessary to promote net fluxes of water across the plasma membrane, non-selective Ca^2+^-permeable mechanosensitive channels (MSCs) have also been implicated in the regulation of cell volume [8, 15, 83, 86, 124, 161]. During hypotonic cell swelling, MSCs can sense changes in plasma membrane tension/stretch and activate as result. Their activation then triggers intracellular Ca^2+^ signals that in turn regulate various effectors, such as Cl^−^ and K^+^ channels. However, the involvement of Ca^2+^ in the RVD is rather controversial and unclear, as evidenced by the conflicting data reported in the literature.

### The role of Ca^2+^ in cell volume regulation

In several cell systems, exposure to hypotonic stimuli triggers a rise in intracellular Ca^2+^ concentration, as the result of both Ca^2+^ influx from extracellular space through MSCs [8, 15, 19, 83, 86, 96, 124, 161] and its release from internal stores [99, 127, 149]. Despite the ubiquity of cytosolic Ca^2+^ transients evoked by hypotonic cell swelling, intracellular Ca^2+^ signals are not always needed for the RVD response. Indeed, while in certain cell preparations, RVD occurs independently of cytosolic Ca^2+^ mobilization [13, 18, 128], other cell types strongly require Ca^2+^ [71, 73, 87, 111]. The contribution of Ca^2+^ in RVD likely depends on the specific set of Cl^−^ and K^+^ channels expressed in different cell types.

#### Ca^2+^ and the activation of VRAC

Whether VRAC is regulated by Ca^2+^ has been controversial since its discovery in the late 1980s. It is generally assumed that VRAC activation is a Ca^2+^-independent phenomenon and that the main trigger of the channel is the reduction of intracellular ionic strength, as well as the stretch of the plasma membrane following exposure to osmotic stress [23, 138, 157]. Consistent with this notion, activation of VRAC by hypotonic cell swelling occurs also in conditions of heavy intracellular Ca^2+^ buffering, as well as in the absence of cytosolic Ca^2+^ increase [2, 26, 74, 113]. In line with these data, in human glioblastoma (GBM) cells, we found that VRAC-mediated I_Cl,swell_ is under the control of a PLC-dependent signalling pathway activated by the hypotonic stimulus and Ca^2+^-independent, as it is not affected by the Ca^2+^ chelator BAPTA [26]. Conversely, in other cell types, cytosolic Ca^2+^ signals are needed for both VRAC-mediated I_Cl,swell_ activation, and RVD [3, 14, 19, 89, 90, 99, 147], although the underlying mechanisms are generally unknown. In HEK293 cells, however, we have reported that VRAC-mediated I_Cl,swell_ can be modulated by cytosolic Ca^2+^ signals generated by activation of the Ca^2+^-induced Ca^2+^ release (CICR) mechanism [147].

#### Ca^2+^ and the activation of anoctamins (ANO) channels

In some cell types, the sensitivity of the RVD process to Ca^2+^ depends on a significant expression of Ca^2+^-activated anion channels ANO1 and ANO6, also known as TMEM proteins [6, 75, 160]. Their activation generates outward-rectifying Cl^−^ currents requiring intracellular ATP and activated by osmotic cell swelling following the entry of extracellular Ca^2+^ [16]. ANO1/6-mediated currents are distinguished from VRAC currents by the absence of voltage-dependent inactivation, insensitivity to VRAC blocker DCPIB, and sensitivity to ANO inhibitors T16Ainh-A01 and CaCCinh-A01 [41, 142]. Almaça and coworkers found that ANO1 knock-down significantly reduced the I_Cl,swell_ and the RVD in the presence of extracellular Ca^2+^ in epithelial cells [6]. Moreover, ANO6 knock-out mice show altered RVD in murine submandibular salivary glands [120].

#### Ca^2+^ and the activation of IK and BK channels

The Ca^2+^ dependence of RVD is also related to the type of K^+^ channels expressed in a specific cell type. In general, epithelial cells expressing K_Ca_ channels exhibit a Ca^2+^-dependent RVD response, whereas in non-epithelial cells, where other K^+^ channels are expressed (i.e., stretch-activated and voltage-dependent K^+^ channels), the RVD is largely Ca^2+^ independent [63, 128]. As for K_Ca_ channels, several studies have reported the involvement of both IK and BK channels in the RVD response [53, 166, 169]. However, the mechanism by which these channels are activated by cell swelling is still under investigation. Both IK and BK channels require an elevation of intracellular Ca^2+^ to open, despite they differ markedly for their sensitivity to Ca^2+^. While IK channels have a high affinity for Ca^2+^ (EC_50_: 100–200 nM) [50], BK channels exhibit a significant lower sensitivity (EC_50_: 1–5 µM) [56, 155]. During cell swelling, global cytosolic Ca^2+^ levels have been reported to raise up to 400 nM [121, 170, 174], high enough to activate most IK channels, but insufficient to open BK channels at the resting membrane potentials, making the hypotonic activation of BK channels controversial. On this ground, some studies have even concluded that BK channels can be activated directly by membrane stretch [5, 31, 108, 159]. We will return to discuss the activation of BK channels induced by hypotonic stimuli once we have acquired more information on mechanoreceptors and specific sub-membrane structures. For the time being, we conclude this section by saying that, regardless of whether or not it is required for the activation of the RVD process, increases in cytosolic Ca^2+^ following hypotonic stress are the result of Ca^2+^-permeable MSCs that convert and are gated by, forces applied to the plasma membrane. Therefore, it is plausible to infer that the activation of both BK and IK channels is under the control of the stretch-induced Ca^2+^ entry through MSCs. Consistent with this notion, it has been reported that the removal of external Ca^2+^ or the presence of gadolinium (Gd^3+^), a potent inhibitor of MSCs, inhibits the hypotonic activation of BK channels and significantly impairs the RVD process [77].

#### MSCs and cell volume regulation

MSCs form a large family of evolutionarily ancient channels, present in all animal kingdoms. They function as molecular transducers of mechanical stimuli for all kinds of sensory functions, proprioceptive signals, and in the control of cell volume. They belong to several families of channels that differ in distribution, structure, gating, and for the ion species they allow to pass. Since their discovery in invertebrates as the main MSCs, members of the transient receptor potential (TRP) superfamily, mainly TRPV4 and TRPM7 channels, have been postulated to mediate mechanotransduction in cell volume regulation in vertebrate cells [8, 15, 83, 86, 96, 124, 161]. However, inconsistent data from different studies still leave the molecular nature of the Ca^2+^-permeable MSCs uncertain. However, evidence for their role in Ca^2+^ signalling in association with cell volume regulation in vertebrates is scarce. The other important classic family of mechanosensitive K^+^-selective channels is the two-pore-domain K^+^-channel family, with each channel subunit made of four transmembrane segments, which includes the TREK subfamily proteins, comprising TREK-1, TREK-2, and TRAAK [11, 106, 107, 122, 129, 130]. These channels also lack strong evidence of their involvement in cell volume regulation.

In 2010 a new family of MSCs—the Piezo channel family composed by two members Piezo1 and Piezo2—was reported and opened unexpected outlooks in mechanotransduction and Ca^2+^ signalling. Piezo channels display all the features of mechanosensitivity and respond to a variety of mechanical stimuli, including membrane stretch and cell swelling, which lead to opening the nonselective cationic pore that also lets Ca^2+^ ions through. To link Piezo channels more tightly with the topic of this review, we recall that purified Piezo channels reconstituted into lipid bilayers have been found to generate osmolarity-sensitive currents [156]. In addition, Piezo1-mediated Ca^2+^ influx was reported to be essential for the regulation of cell volume in erythrocytes thanks to the modulation of IK channels [21]. More recently, Piezo1 has been shown to control the RVD process and cell migration in HEK293 cells [147]. In human GBM cells, the same laboratory also provided evidence that the activation of both IK and BK channels upon the hypotonic stimulus occurs as the result of Ca^2+^ influx through Piezo1 and that their activation is essential for the RVD process [114]. These observations make the Piezo1 channel family especially important in the regulation of cell volume, and to learn about the mechanisms underlying this regulation, we now describe in detail its structure, gating, and biophysical properties.

### Piezo1 channel structure, gating, and biophysical properties

**Fig. 2:**
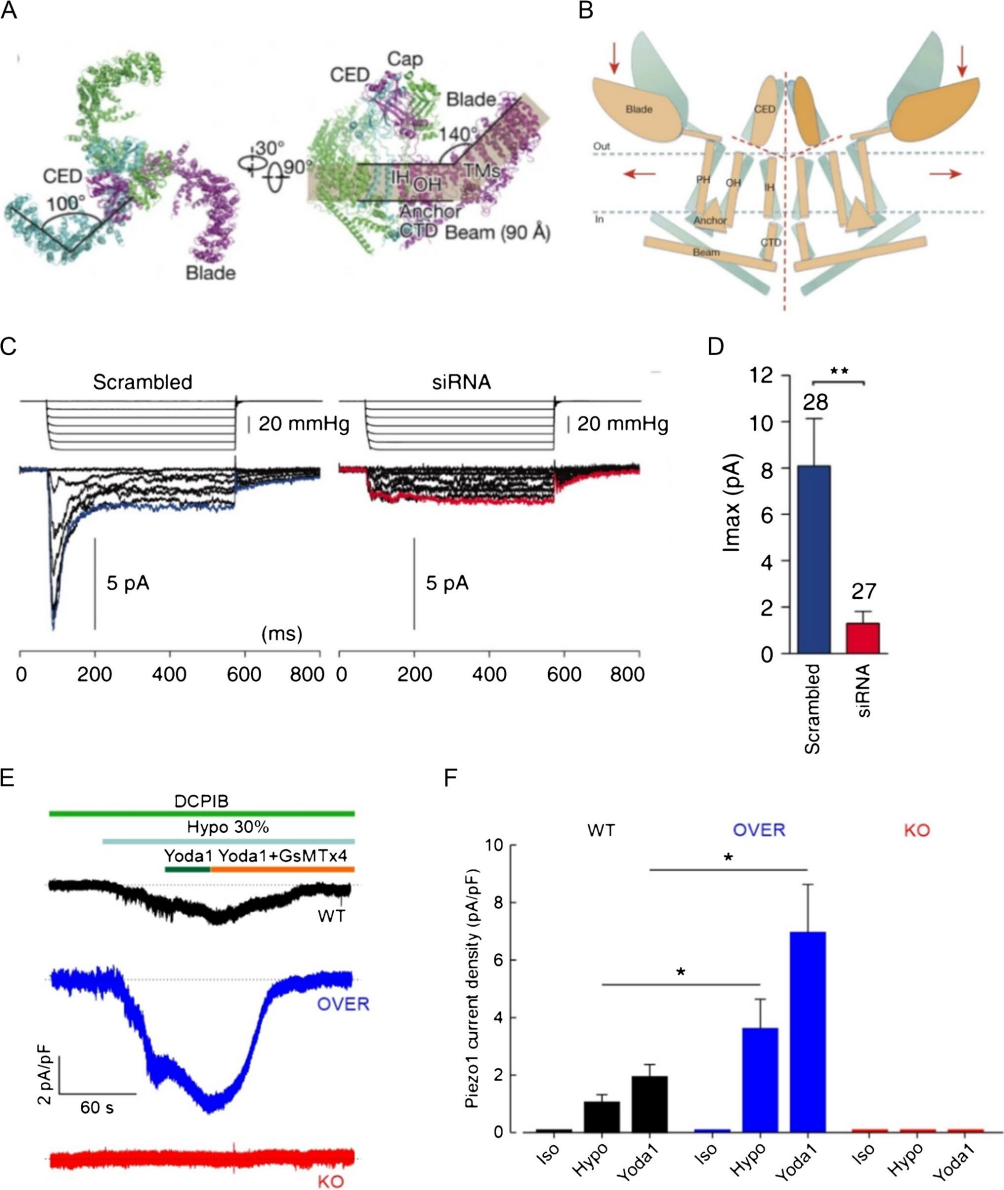
Structure, gating, and biophysical properties of Piezo1 channel. **A** Different views of the trimeric Piezo1 structure with the major domains labelled and the three subunits shown in different colors. Adapted from [178]. **B** Model of the “lever-like mechanotransduction model” of Piezo1 channel. Adapted from [60]. **C** Representative current traces elicited by applying a negative pipet pressure in N2A cells transfected with (left) scrambled siRNA or (right) Piezo1 siRNA. Traces of current elicited by − 60 mmHg are highlighted in blue and red for scrambled siRNA and Piezo1 siRNA, respectively. **D** Bar plot showing the maximal amplitude of stretch-activated currents elicited at a holding potential of − 80 mV in N2A cells transfected with scrambled siRNA (blue) or Piezo1 siRNA (red). Data are shown as mean ± SEM. Adapted from [34]. **E** Representative whole-cell Piezo1 current at − 80 mv in WT, Piezo1 overexpressing (OVER) or knockout (KO) HEK293 cells, elicited by exposure to extracellular 30% hypotonic solution. **F** Bar plot showing Piezo1 currents under control conditions (Iso) and following application of either hypotonic solution (Hypo) or the selective Piezo1 agonist Yoda1 (Yoda 1), in WT, OVER and KO HEK293 cells. Adapted from [147]

Piezo1 is a large nonselective channel permeable to both monovalent and divalent cations, composed of approximately 2500 amino acid residues [34]. Cryo-electron microscopy studies of the full-length protein revealed that Piezo1 channel is a homotrimeric complex, with each subunit containing up to 38 transmembrane domains [140, 178] (Fig. 2A). The channel exhibits a triple-blade propeller structure and a central ion-conducting pore formed by a C-terminal domain (CTD) and a C-terminal extracellular domain (CED). The extracellular “cap” domain, localized at the top of the central axis [60, 65, 140], is formed by the CED of each subunit. The distal regions of the three blades communicate directly with the central pore through beam domains, helical structures forming a 30° angle with the plane of the membrane and proposed to couple blade conformation to pore gating. In the closed state, Piezo1 protein, including the lipid bilayer encircled by the channel’s perimeter, appears in the form of a nanodome more than 20 nm in diameter and 6–9 nm in depth [65, 68–70]. The 38 transmembrane helices, unusually bent relative to the plane of the membrane, favor a prominent localized curvature of the membrane, which appears to confer the extraordinary mechanosensitivity of the channel. Only the three-blade subunits would participate in this, but not the central pore [123].

Concerning the gating of the channel, structure–function studies indicate that Piezo1 is directly opened by membrane stretch in the absence of other cellular components, suggesting that it directly senses forces from membrane lipids, such as lateral tension and curvature [65, 93]. This exceptional mechanosensitivity can be explained by the structure/architecture of the Piezo1 channel, which led to the hypothesis of a “lever-like mechanism” for its opening during membrane deformation (Fig. 2B). This type of Piezo1 activation, explained by the “lateral membrane tension model” suggests that the membrane stretching would promote a transition of blade domains from the curved to the flattened state. This conformational change would turn the beam domain as a lever that uses the L1342 and L1345 residues as pivot, resulting in the opening of the central pore [36, 42, 97]. This model is supported by several lines of evidence showing that forces coming from intact membrane are sufficient *per se *to open Piezo1 [35]. In addition, direct fluorescence nanoscopy has recently demonstrated that an increase in membrane tension increases the distance between the distal points of the three blades, in accordance with the acquisition of a flattened conformation [119]. However, other studies support an alternative gating mechanism for Piezo1 opening, exemplified by the “tethered spring model,” in which Piezo1 is activated through interaction with the cytoskeleton or the extracellular matrix components [59, 167, 171].

In patch clamp electrophysiology, the two main mechanical stimuli used for Piezo1 activation are membrane indentation, obtained by applying positive pressure to the cell surface through a fine glass probe in whole-cell configuration, and membrane invagination, obtained by applying negative pressure through the patch micropipette in the cell-attached configuration [34, 62]. However, Piezo1 activation can also be triggered by other physiologically relevant mechanical stimuli such as membrane stretch, flow shear stress, and osmotic stress [34, 134, 156, 179]. The unitary conductance recorded in cell-attached configuration is ~ 35 pS, with a reversal potential near 0 mV and a linear current–voltage relationship in a voltage range between − 80 and + 80 mV. Another important feature of Piezo1 biophysics is its rapid inactivation upon membrane indentation in whole-cell configuration, with an inactivation time of approximately 15 ms at − 80 mV [34] (Fig. 2C and D). Gottlieb et al. proposed a linear three-state model of closed, open, and inactivated, to fit the kinetic properties of Piezo1 gating [62]. Notably, *PIEZO1* mutations in the pore and extracellular CAP region that cause dehydrated hereditary stomatocytosis, a genetic condition with an imbalance in intracellular cation concentrations, give rise to mechanically activated currents that inactivate more slowly than wild-type currents, resulting in a gain of function of channel activity [4]. In accordance, additional mutations in the extracellular CAP and inner helix pore domain were found to affect the Piezo1 channel inactivation kinetics [94, 172].

Piezo1 channels are permeable to monovalent (K^+^, Na^+^, and Cs^+^) and divalent (Ba^2+^, Ca^2+^, and Mg^2+^) cations, with a selectivity sequence of Ca^2+^  > K^+^  > Na^+^  > Mg^2+^ [34]. Thus, the Ca^2+^ influx through Piezo1, induced by mechanical stimulation, modulates several cytoplasmic signalling pathways involved in different physiological processes such as proliferation, apoptosis, and migration [28, 30, 32, 82, 173]. All these cellular processes require the ability of cells to finely regulate their volume and shape, a mechanism that is under the control of several membrane transporters and in which a growing body of research is showing Piezo1 to be involved deeply [21, 114, 147].

### Piezo1 as a key player in cell volume regulation

For a cell to regulate its volume, it is necessary that it can detect volume changes and use this information to trigger feedback mechanisms that bring back the cell volume to the original condition. Notably, among the many different types of mechanical stimuli, Piezo1 displays a significant sensitivity also to changes in the overall volume of the cell, thus being a candidate sensor for the process of volume regulation (Fig. 2E and F). In fact, Piezo1 activity increases upon hypotonic-induced cell swelling in a variety of cells, such as urothelial cells [116], rat beta cells [43], colangiocytes [44], bladder interstitial Cajal-like cells [98], and HEK293 cells heterologously expressing Piezo1 [147]. In addition, conformational changes of the blades associated with Piezo1 channel activation have been found to be induced by cell swelling [119].

Since their discovery in 2010, strong attention has been placed on Piezo channels, especially Piezo1, as the main mechanotransducers underlying cell volume regulation.

The idea of Piezo1 being the Ca^2+^ permeable mechanosensitive channel involved in cell volume regulation, started with the identification of gain-of-function mutations in *PIEZO1* gene linked to human hereditary disorders affecting erythrocytes, known as dehydrated xerocytosis and stomatocytosis. Both disorders are characterized by defective membrane properties that enhance cation permeability and alter cell volume homeostasis [4, 9, 177]. Erythrocytes expressing gain-of-function mutation of the Piezo1 channel, are indeed characterized by a decreased intracellular cation concentration which in turn promotes the efflux of osmotic water and a reduction of erythrocytic cell volume (i.e., dehydration). This strongly indicates that Piezo1 controls the erythrocyte volume, although the exact molecular mechanism remains unclear. The link between mechanical forces and volume regulation by Ca^2+^ influx through Piezo1 has been clearly demonstrated by Patapoutian and colleagues and others, showing that Piezo1 controls the activity of IK channels, and the consequent efflux of K^+^ ions generates the osmotic gradient for the net water loss [21, 38]. The resulting reduction of the erythrocyte volume makes it possible for them to pass through small-diameter capillaries. In accordance with this notion, *PIEZO1* knockdown results in a drastic reduction in circulating erythrocytes, which appear spherical and swollen with signs of membrane ruptures [51]. However, the mechanisms underlying cell volume control during the passage of erythrocytes through capillaries is still debated. A recent modelling study reveals an unexpected up-down biphasic volume response during the passage of erythrocytes through capillaries, characterized by an initial tiny but sharp increase of cell volume, followed by a slow shrinkage towards below-baseline volume levels [135].

**Fig. 3:**
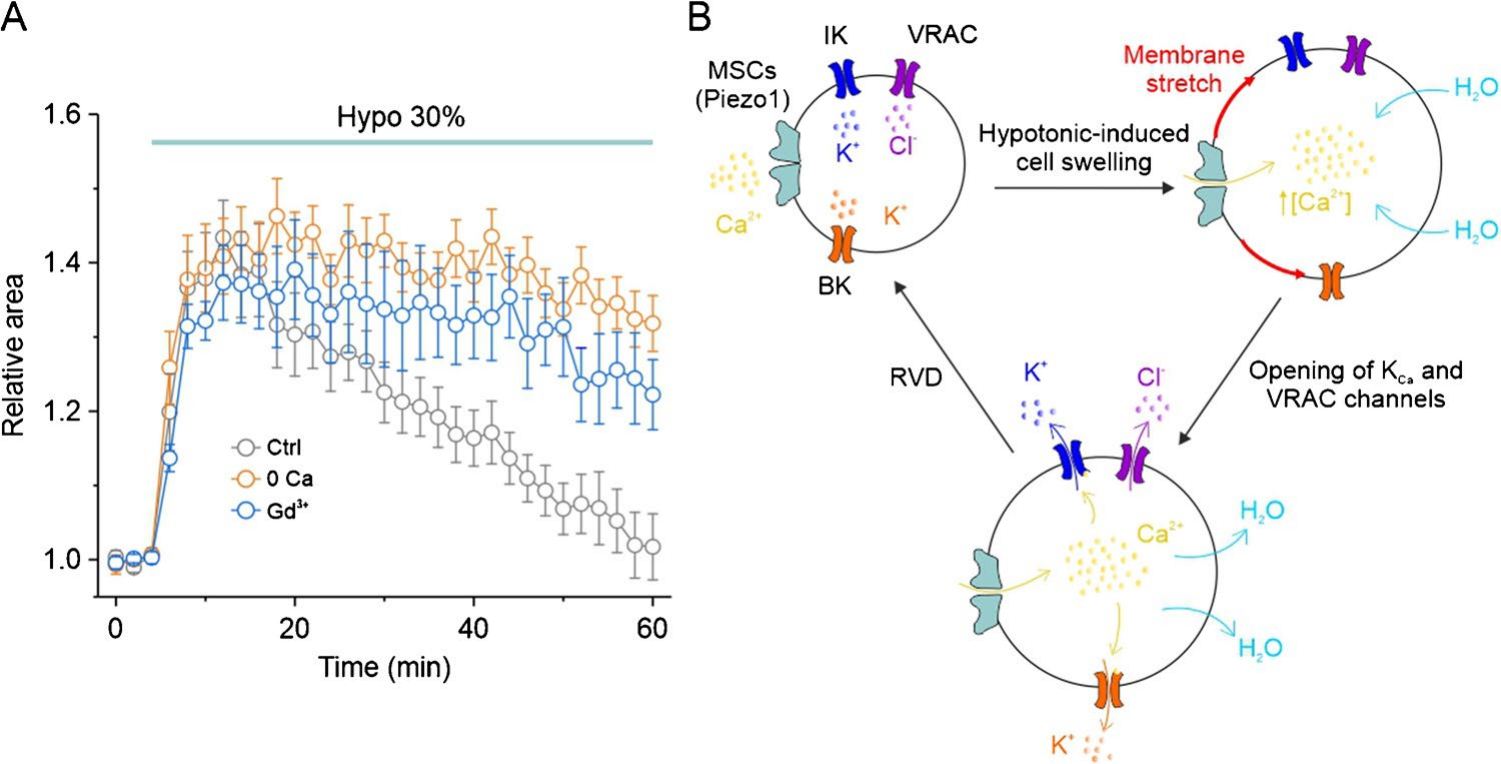
Possible involvement of Piezo1 in cell volume regulation (RVD) of human GBM cells. **A** Time course of RVD under various experimental conditions (control, zero external Ca^2+^, external Gd^3+^) observed in response to the application of 30% hypotonic solution. Data are shown as mean ± SEM. **B** Schematic illustrating the proposed mechanisms underlying the osmotic stress-induced RVD process. The influx of extracellular Ca^2+^ through MSCs, including Piezo1, is an essential step for the activation of K_Ca_ channels and, by consequence, for the RVD. Panel B, adapted from [114]

In human GBM cells, we recently reported that the influx of Ca^2+^ through MSCs activated by hypotonic cell swelling is a key prerequisite for the activation of both IK and BK channels, which are necessary for the occurrence of RVD [114] (Fig. 3). An important observation of this study is that a current very similar to the hypotonic activated current, exhibiting the biophysical (i.e., reversal potential close to 0 mV and strong outward rectification) and pharmacological (i.e., block by Gd^3+^) properties of nonspecific mechanosensitive cation MSCs, is observed under isotonic conditions upon application of the highly selective Piezo1 agonist Yoda1. In addition, this molecule also activates IK and BK channels in the absence of cell swelling. These data strongly indicate that Piezo1 is the main component of MSCs activated by cell swelling in GBM cells and the main responsible for mechanotransduction during GBM cell volume regulation. It is worth noticing that Piezo1 expression levels increase with the grade of gliomas [29, 132, 133]. This observation makes Piezo1 an excellent candidate as the main MSC involved in GBM cells volume regulation. However, to date, there is insufficient information to support this conclusion and further studies will be necessary.

However, it remains unclear how the relatively small increase of global cytoplasmic Ca^2+^ concentration (300–400 nM), induced by cell swelling, can activate BK channels at physiological membrane potentials. To overcome this apparent inconsistency and for BK channels to sense micromolar concentrations of Ca^2+^, they should either co-localize with mechanosensitive Ca^2+^ sources (i.e., Piezo1 channels) or be confined to specialized compartments of the plasma membrane associated with intracellular Ca^2+^ stores (i.e., endoplasmic reticulum), where the Ca^2+^ signal can be amplified by activation of the CICR mechanism. Notably, both prospects have been reported in several cell types. In neurons, where BK channels play a key role in the regulation of action potential duration, K^+^ channels are physically associated with voltage-dependent Ca^2+^ channels and thus immersed in their Ca^2+^ microdomains where they sense up to hundreds of micromolar Ca^2+^ [48, 49, 110, 148, 164]. Conversely, in other cell types, BK channels are confined together in specialized sub-compartments of the plasma membrane, such as lipid rafts or caveolae, associated with the endoplasmic reticulum. In these micro-compartments, BK and Ca^2+^-permeable channels in the plasma membrane are not physically associated, and the elevation of Ca^2+^ to levels sufficiently high to activate BK channels at physiological voltages is ensured by the CICR mechanism [10, 72, 150, 154, 168]. A functional, non-physical coupling between Piezo1 and BK channels has been reported in different cell types, such as fibroblasts and epithelial cells [57, 78]. However, more in-depth studies are required to unravel the precise mechanisms by which BK channels are activated by the influx of Ca^2+^ during cell swelling and participate in the regulation of cell volume.

A direct demonstration of the role of Piezo1 in the regulation of cell volume has also been reported in a study showing that the ability of HEK293 cells to restore their volume upon osmotic shock-induced cell swelling (RVD) is strongly correlated with Piezo1 expression [147]. At the molecular level, the mechanism by which Piezo1 modulates cell volume in HEK293 cells involves the modulation of VRAC‐mediated I_Cl,swell_, through the CICR mechanism, which is necessary to fully activate the hypotonic-stimulated VRAC channels. However, given the Ca^2+^ independence of VRAC‐mediated I_Cl,swell_ [14, 26, 27], the exact mechanism by which Piezo1-mediated elevation of intracellular Ca^2+^ levels regulates VRAC channels has remained unexplained. One possibility would be that HEK293 cells express Ca^2+^-sensitive proteins that positively modulate the activity of VRAC. At confirmation of this notion, our recent publication reported that expression of the astrocyte-specific MLC1 protein confers Ca^2+^-sensitivity to the otherwise insenitive VRAC [19].

Piezo1 has also been shown to control cell volume in smooth muscle cells. Specifically, Piezo1 activation in the rigid extracellular matrix has been shown to increase the cell volume of vascular smooth muscle cells, exacerbating aortic wall rigidity, and decreasing aortic compliance. This effect is mediated by an increase in membrane water permeability following Piezo1-induced Ca^2+^ influx and consequent activation of PKC, which promotes membrane expression of aquaporins [80]. Together, these results underscore the crucial role of Piezo1 in the mechanotransduction process associated with cell volume regulation in different cell types.

Although studies by us [147] and by the Patapoutian’s group [21] unequivocally demonstrated that Piezo1 is the MSC directly responsible for the regulation/control of cell volume, it is important to underline that other works argued against this vision. One essential example is the early paper by the Sachs laboratory, showing that the potent Piezo1 blocker GsMTx4 inhibits cell volume regulation in normal kidney NRK-49F cells but not in MDCK cells or primary rat astrocytes [76]. In the same study, the authors demonstrate that Gd^3+^ blocks RVD in a manner that is independent of Piezo1. These data suggest that the role played by Piezo1 in cell volume regulation strongly depends on cell type. In addition, the involvement of Piezo1 in physiological processes cannot be based exclusively on the use of Gd^3+^.

### Physiopathological role of Piezo1 in relation to its ability to control cell volume

In this section, we will first present evidence conclusively showing that Piezo1-related diseases depend on its lost ability to regulate cell volume. We will then describe other demonstrated roles for this channel in which the involvement of Piezo1-mediated cell volume regulation is likely to be involved.

#### Piezo1 and erythrocytes

As described in the previous paragraph, the role of Piezo1 in human physiopathology emerged from studies on erythrocytes. Throughout their lives, erythrocytes have to reshape their profile and reduce their volume (squeeze) to pass through capillaries with diameters half their own. Piezo1 is essential in this mechanotransduction process that allows osmotic reduction of erythrocyte volume. It has been shown that erythrocytes exhibit robust Ca^2+^ entry in response to mechanical stretch and this entry is dependent on Piezo1 expression. Furthermore, erytrocytes from Piezo1 knockout mice are overhydrated and exhibit increased fragility both in vitro and in vivo. The ability of Piezo1 to control erythrocyte volume relies on the downstream activation of the IK channel and the subsequent efflux of osmolytes followed by osmotic water [21]. Accordingly, several mutations causing a gain of function of Piezo1 have been associated with hereditary xerocytosis, a rare disease associated with erythrocyte dehydration [7, 61, 177].

#### Piezo1 and arterial smooth muscle

Decreased aortic compliance, caused by an increased rigidity of the aortic wall and the vascular smooth muscle cells, is a precursor to numerous cardiovascular diseases. During aging, arterial wall rigidity is caused by extracellular matrix stiffening, which enhances contractile forces produced by vascular smooth muscle cells [1, 81]. Notably, vascular smooth muscle cells significantly express Piezo1 channels [102], which in turn promotes Ca^2+^ influx and subsequent activation of PKC and aquaporin-1 plasma membrane expression [80]. Interestingly, increased Piezo1 activity in aging vascular smooth muscle cells is also responsible for the reduced mechanosensitivity of the vasculature [104] and arterial calcification observed in many vascular pathologies [158]. Pharmacological targeting of the Piezo1/PKC/aquaporin-1 pathway can thus be used to prevent the vascular smooth muscle cell volume response induced by matrix rigidity, as well as the reduced mechanosensation and increased calcification observed in pathological conditions. Importantly, upregulation of both Piezo1 and aquaporin-1 gene expression is observed in disease-relevant vascular smooth muscle cell phenotypes [80].

#### Piezo1 and tumors

A growing body of evidence in recent years has linked the aberrant functional expression of Piezo1 to tumor malignancy in different types of cancer [175]. Tumor cells are characterized by uncontrolled proliferation, dedifferentiation, resistance to cell death, and a high migratory and invasive potential. Many studies have recently involved Piezo1 in cell migration and invasion in tumors [95, 103, 153, 175, 176].

In gliomas, Piezo1 expression has been shown to correlate closely with the tumor malignancy grade and inversely with patients’ survival [133, 180]. GBM, the most aggressive and lethal primary adult brain tumor, showing the highest malignancy among gliomas (IV grade), expresses Piezo1 at the highest level. GBM has a very poor prognosis and a median survival of 15 months because of the high rate of infiltration into the peritumoral parenchyma by isolated tumor cells, leading to tumor regrowth outside the central mass [33, 39, 40]. This occurrence restricts greatly the success of surgical resection and exacerbates the prognosis. The invasion of GBM cells into the narrow spaces of the healthy brain parenchyma requires their ability to sense the presence of external obstacles and forces from the environment and adjust their volume and shape accordingly to pass through. Currently, the molecular determinants of this process are mostly unknown.

It is now widely accepted that both VRAC and K_Ca_ channels are essential for osmotic volume regulation underlying the migratory/invasive processes of GBM cells [25, 37, 64, 85, 144]. In contrast, little is known about the role played by MSCs, such as Piezo1, in GBM cell migration/invasion. However, we recently reported that both BK and IK channels, which are widely recognized as the major K^+^ channels of GBM cells involved in migration/invasion processes [137, 151, 162], are under the control of Piezo1 and that the Piezo1-BK/IK coupling is essential for the regulation of the cell volume [114]. Therefore, the mechanosensitive Ca^2+^-permeable Piezo1 channel could be used by GBM cells to sense mechanical stimuli from the tumor microenvironment, in the form of local deformation of the plasma membrane (i.e., stretch, indentation, invagination etc.), and to induce an increase in cytoplasmic Ca^2+^ that enables cell volume and shape modifications necessary for the cell to invade the healthy brain parenchyma, through activation of K_Ca_ channels and regulation of actin cytoskeletal polymerization.

Similarly, mechanosensitive Ca^2+^-permeable channels including Piezo1 have been found crucial for the malignancy of prostate cancer. In this regard, Maroto and colleagues initially reported that the block of MSCs channel in highly migratory/invasive PC3 prostate tumor cells, with both GsMTx-4 and Gd^3+^, significantly blocks both elevations in intracellular Ca^2+^ concentrations and cell migration [109]. Interestingly, the influx of Ca^2+^ through MSCs promotes the activation of SK channels. The link between MSCs and one of the three members of K_Ca_ channels, found in PC3 cells, is quite similar to that reported by us in GBM cells [114], suggesting a common mechanism used by tumor cells to migrate/invade. However, neither the study by Maroto nor that by us unequivocally demonstrate that Piezo1 is the only MSC involved. In the first case, in fact, genetic suppression, or overexpression of specific members of TRPC1 and TRPC3 channels inhibit PC3 cell migration, although MSC activity remained unaltered, suggesting that these channels may do so using other mechanisms different from mechanotransduction. Notably, though, other studies reported that Piezo1 is upregulated in prostate tumor cell lines such as DU145 and PC3 cells, as well as in human prostate tumor biopsies, where it plays a crucial role in the epithelial-to-mesenchymal transition essential for tumor progression [67, 101]. Future studies will be essential to shed light on this scientific issue. Regarding our study on GBM cells, we only used a pharmacological approach (i.e., the use of Gd^3+^), which cannot distinguish among several types of MSCs. A genetic approach via the ablation of *PIEZO1* gene will therefore be necessary to unequivocally demonstrate that Piezo1 represents the main MSC responsible for GBM cell volume regulation. 

#### Piezo1 and cell migration

Piezo1 has been found to be involved in many different physiological and pathological processes where cell migration is fundamental, such as skin wound healing [28, 182], cancer metastasis [46], immune response of brain microglia [181], and cell spreading on micro-patterns [79]. Although the precise mechanism through which Piezo1 exerts its role in cell migration is still mostly unknown, we propose that cell volume regulation is surely involved.

The link between Piezo1’s ability to control cell volume and its potential role in cell migration can be inferred from the classical view of this process. Cell migration is a multistep process that requires a front-rear polarization of the cell and can be divided into two main phases: (i) protrusion of the front edge and adhesion to the extracellular matrix via the assembly of focal adhesions and (ii) disassembly of focal adhesions and consequent retraction of the rear edge [115]. Protrusion of the front edge is mediated by the cytoskeletal actin polymerization and assembly of focal adhesions that allows the cell to interact with the extracellular matrix. This phase requires an increase in cell volume, which is warranted by the net uptake of solutes, mainly Na^+^, K^+^, and Cl^−^, and the consequent osmotic influx of water. Among the membrane transporters involved in the osmotic increase of cell volume in the front edge, the Na^+^/K^+^/2Cl^−^ cotransporter (NKCC1) plays an essential role [105, 143, 152]. Retraction of the rear edge is, instead, brought about by the net efflux of KCl mediated by Cl^−^ and K^+^ channels, which is followed by osmotic loss of water and the resulting decrease of cell volume. MSCs are thought to be involved in both phases, due to forces acting on the plasma membrane. During the front edge protrusion, the assembly of focal adhesions necessary for the interaction of the cell membrane with the extracellular environment prompts local increase of membrane tension or the recruitment of Piezo1 channels in situ. Ca^2+^ influx through mechanically activated Piezo1 channels is the signal for further actin cytoskeletal polymerization and consolidation [22, 100]. Piezo1 could also be crucial in the retraction of the rear edge. Piezo1 channels opening in this region of the cell would be triggered by membrane stretching caused by the increased volume of the front edge. The consequent influx of Ca^2+^ would promote both the actomyosin-dependent disassembly of focal adhesion [117] and the activation of K^+^ channels such as K_Ca_, essential for the osmotic loss of water, the local reduction of cell volume, and the consequent retraction of the rear edge.

Therefore, Piezo1 channel could be used by cells to sense mechanical stimuli from the environment and induce an increase in cytoplasmic Ca^2+^ that enables cell volume and shape modifications necessary for the cell to progress, through activation of K_Ca_ channels and regulation of actin cytoskeletal polymerization.

#### Piezo1 and cell death

Recent studies have demonstrated that Piezo1 plays an important role in Ca^2+^-dependent cell death induced by mechanical stimuli [82]. In human articular chondrocytes, exposure to hydrostatic pressure leads to increased Ca^2+^ levels and upregulation of p53 expression and caspase-3 and -9 cleavage, hallmarks of apoptotic death. Notably, Gd^3+^ or Piezo1 knock-down prevents these events [88]. Similarly, the activation of Piezo1 by mechanical pressure leads to chronic death of pancreatic acinar cells, ultimately responsible for pancreatitis [136]. Although the precise mechanism by which Piezo1 controls cell death is still unknown, there is the possibility that its ability to control cell volume is involved. Indeed, volume changes are crucial in several forms of cell death, such as in apoptotic death, which is preceded by a contraction of cell volume known as apoptotic volume decrease (AVD) [17, 125]. In GBM cells, K_Ca_ channels are directly involved in AVD caused by the addition of either staurosporine or TNF-α-related apoptosis-inducing ligand (TRAIL), which activate the intrinsic or extrinsic pathway of apoptosis, respectively [112]. Hence, a general pattern for AVD could be a volume reduction as result of water efflux following activation of K_Ca_ and VRAC channels consequent to Piezo1 activation by mechanical stimulation.

Conversely, an increment of cell volume is observed during necrosis and is called necrotic volume increase (NVI) [12, 125]. This is mainly caused by the influx of NaCl, due to the lack of energy supply and the reduced activity of the Na^+^/K^+^ pump, followed by osmotically driven entry of water. It could be speculated that mechanically-induced Ca^2+^ entry could disfavor NVI and prevent necrotic cell death in cells expressing K_Ca_ channels that are activated by raises of cytosolic Ca^2+^ levels, but further studies are needed to conclusively clarify the relationship between necrosis and Piezo1 activity.

The proper regulation of cell volume is also an important aspect for erythrocytes physiology, such as senescence. It is well known that senescent erythrocytes are characterized by a series of changes including cell dehydration and loss of deformability that precede their removal from the circulation by the spleen [84]. The process whereby aged erythrocytes become dehydrated and undeformable is known as the “Gardos effect” as it mainly involves the IK channel, known as the Gardos channel in erythrocytes, as firstly identified in these cells by Gardos in the late fifties [58]. It has been reported that the increase of intracellular Ca^2+^ necessary for the activation of the Gardos channel occurs upon ATP depletion-dependent proteolysis of outwardly rectifying Ca^2+^ pumps (i.e., PMCA) [84].

However, the required elevation of cytosolic Ca^2+^ necessary for the Gardos channel-mediated K^+^ and water efflux, may be mediated by Piezo1, as unambiguously observed in experiments using Piezo1 activator Yoda1 and Piezo1 inhibitor GsMTx4 [91].

Interestingly, the first patch-clamp recordings by Hamill, reported two classes of K_Ca_ channels (IK and SK) that, in addition to a volume-activated outward rectifying Cl^−^ channel, are responsible for the Gardos effect [66]. In this work, Hamill shows that IK channel is more sensitive to Ca^2+^ applied to the inside-out patch, whereas SK channel is more readily activated by cell swelling. Given the activation of Piezo1 by the hypotonic stimulus, these results would strengthen the hypothesis of its involvement in the Gardos effect in erythrocytes.

## Conclusions

Data accumulated over the past fifteen years have described the mechanosensitive and Ca^2+^-permeable Piezo1 channel as the main sensor of physical forces on the plasma membrane and transducer of mechanical stimuli into intracellular Ca^2+^ signals underlying several biological processes, such as proliferation, migration, and cell death. The modulation of these processes have been attributed to Piezo1-mediated Ca^2+^ activation of multiple intracellular pathways.

In this review, we have reported more recent data that introduce another mechanism by which Piezo1 activation by membrane forces can regulate proliferation, migration, and cell death. In this new perspective, Piezo1 plays the main role in these processes by regulating the cell volume. In GBM cell models, in which these processes assume a particular importance, Piezo1-mediated Ca^2+^ influx has been shown to regulate cell volume by activating the K_Ca_ channels IK and BK, which, together with VRAC, are responsible for cell volume control. Previous data demonstrating that IK and BK channels are critical for cell migration in GBM cells and that Piezo1 expression on HEK293 cells finely correlated with cell migration, strongly support the idea that Piezo1 modulates migration by controlling cell volume through regulation of both K_Ca_ and VRAC channels. A similar argument can be made for the fact that Piezo1 controls apoptotic cell death in GBM through the regulation of K_Ca_ channels and ultimately cell volume. In our view, the available data are now quantitatively and qualitatively more than sufficient to include the following chain mechanism of Piezo1 activation Ca^2+^ influx -> IK/BK channels activation -> cell volume regulation as an established biochemical axis in the regulation of proliferation, migration, and cell death by Piezo1.

We have reported convincing data on the Piezo1-centric biochemical axis illustrated above for only some of the biological processes listed earlier and only in some cell models. However, since Piezo1 is expressed in virtually all animal cells and plays an important role in all those processes, the biochemical axis described above can well be considered general in nature.

## Data Availability

Not applicable.

## Abbreviations

*ANO*: Anoctamin
*AVD*: Apoptotic volume decrease
*BK*: Ca^2+^-activated K^+^ channel of large conductance
*CED*: C-terminal extracellular domain
*CICR*: Ca^2+^-induced Ca^2+^ release
*CTD*: C-terminal domain
*GBM*: Glioblastoma
*Gd*^*3+*^: Gadolinium
*I*_*Cl,swell*_: Swelling-activated Cl^−^ current
*IK*: Ca^2+^-activated K^+^ channel of intermediate conductance
*K*_*Ca*_: Ca^2+^-activated K^+^ channels
*MSCs*: Mechanosensitive channels
*NVI*: Necrotic volume increase
*RVD*: Regulatory volume decrease
*SK*: Ca^2+^-activated K^+^ channel of small conductance
*TRAIL*: TNF-α-related apoptosis-inducing ligand
*TRP*: Transient receptor potential
*VRAC*: Volume-regulated anion channel
